# Coronary plaque burden predicts perioperative cardiovascular events after coronary endarterectomy

**DOI:** 10.3389/fcvm.2023.1175287

**Published:** 2023-06-09

**Authors:** Mingxin Gao, Wanwan Wen, Chengxiong Gu, XiaoLi Zhang, Yang Yu, Haiyang Li

**Affiliations:** ^1^ Department of Cardiac Surgery, Beijing Anzhen Hospital, Capital Medical University, Beijing, China; ^2^ Department of Nuclear Medicine, Molecular Imaging Lab, Beijing Anzhen Hospital, Capital Medical University, Beijing, China; ^3^ Department of Ultrasound, Beijing Friendship Hospital, Capital Medical University, Beijing, China

**Keywords:** coronary artery bypass, coronary artery bypass grafting, endarterectomy, myocardial infarction, prognosis

## Abstract

**Background and aims:**

The risk factors of perioperative and long-term cardiovascular events in patients undergoing coronary artery bypass grafting (CABG) with adjunctive coronary endarterectomy (CE) are not well determined. This study evaluated the clinical value of coronary plaque burden, coronary anatomic stenosis, and serum biomarkers for predicting perioperative cardiovascular events after off-pump CABG + CE.

**Methods:**

This retrospective cohort single-center study enrolled 125 patients undergoing off-pump CABG + CE between February 2018 and September 2021 in China. Coronary plaque burden was reflected by the length of plaque removed by CE. Plaque length-max, which represents the plaque length in patients undergoing single-vessel CE and the maximum plaque length in patients undergoing multivessel CE, was calculated. The primary endpoint was perioperative myocardial infraction (PMI).

**Results:**

Plaque length-max was significantly higher in patients with PMI than in those without PMI (2.4 ± 1.5 vs. 1.6 ± 0.9, *p* = .001). A threshold plaque length-max of 1.15 cm was an independent predictor of PMI (area under the curve: 0.67; sensitivity 87.9%; specificity 59.8%; *p* = .005). Patients with plaque length-max ≥1.15 had a > 5-fold increase in PMI after adjusting for confounding factors (odds ratio = 5.89; *p* = .002). Furthermore, interleukin-6 (Beta = .32: *p* = .028), CD68 (Beta = .34; *p* = .045), and osteopontin (Beta = .43; *p* = .008) were significantly correlated with plaque length-max.

**Conclusions:**

Plaque length-max was superior to clinical cardiovascular risk factors in predicting PMI occurrence after off-pump CABG + CE, which might be associated with systemic and plaque inflammation state.

## Introduction

Coronary artery bypass grafting (CABG) is the main surgical intervention for coronary revascularization in patients with multivessel coronary artery diseases; however, it fails to achieve satisfactory outcomes in patients with diffuse coronary artery disease (DCAD) due to incomplete revascularization (1, 2). Coronary endarterectomy (CE), as an adjunct to CABG, has been used to treat patients with DCAD in whom CABG alone could not achieve satisfactory outcomes (3, 4). While the occurrence of perioperative cardiovascular events after CE, including perioperative myocardial infarction (PMI) and mortality, remains up to 19%, which is 2–3 folds higher than that after CABG alone (5–8). These risks of perioperative adverse events are still a major challenge for CABG with adjunctive CE (CABG + CE).

Previous studies have attempted to identify specific biomarkers for predicting PMI (9–11), including serum biomarkers, SYNTAX score, and EUROScore. The prognostic value of serum biomarkers, especially inflammatory biomarkers, such as interleukin-6 (IL-6), tumor necrosis factor, and C-reactive protein, are associated with the occurrence of cardiac events in acute coronary syndrome patients or patients undergoing CABG alone, while these value in DCAD patients after CE is influenced by multi-factors. The endothelial dysfunction or residual lesions induced by coronary plaque removal during CE and vulnerable plaque rupture may all account for cardiac events in patients with DCAD (12–14).

The associations between inflammation, plaque burden, and cardiac events after CE are still unclear. Theoretically, coronary endothelium damage and distal residual lesions may correlate with the length of plaque removed by CE, and plaque length can also reflect the plaque burden of the coronary artery. In addition, pathological analysis of plaques can reveal the inflammatory microenvironment. Therefore, the purpose of this study was to evaluate the clinical value of plaque length, coronary anatomic stenosis (SYNTAX score), and serum biomarker for predicting perioperative cardiovascular events after off-pump CABG + CE.

## Materials and methods

### Study population

This was a retrospective cohort study of 512 patients with coronary artery disease (CAD) who visited the outpatient clinic of Beijing Anzhen Hospital between February 2018 and September 2021. Of these, 143 patients with DCAD were scheduled for off-pump CABG with adjunctive CE (2). Patients with recent myocardial infarction (MI) (within 4 weeks of surgery), history of malignancy, inflammatory or autoimmune disease, or history of cardiac surgery were excluded. Finally, 125 patients were enrolled. The primary endpoint was PMI, which was defined as an elevation of troponin I (TNI) values >10× 99th percentile upper reference limit within 7 days of surgery, in patients together with either: (a) new pathologic Q waves on the electrocardiogram, or (b) new abnormal segment wall motion of the ventricular wall on echocardiography, according to the fourth universal definition of myocardial infarction (2018) by expert consensus.

This study was conducted in accordance with the Declaration of Helsinki. The trial was approved by the Beijing Anzhen Hospital Medical Ethics Committee (No. 2015012X) and was a retrospective sub-analysis of the prospective trial registered with the Chinese Clinical Trial Registry (ChiCTR1900022527). Written informed consent was obtained from all patients without stipend.

### Surgical procedure

Off-pump CABG with adjunctive CE was performed in at least one artery by two experienced cardiologists (CG, YY) if patients with DCAD were unsuitable for off-pump CABG alone (15). Closed-CE involved a smaller arteriotomy than open-CE to remove the atherosclerotic plaque by applying steady, gentle traction on the plaque proximally and distally. If the distal target vessel or its side branches were insufficiently revascularized, a second, concurrent arteriotomy was performed to remove the total distal end of the plaque after angioplasty. Graft flow was measured using the Veri Q flow meter (Medi-Stim Inc., Oslo, Norway) after conventional off-pump CABG to guarantee patency.

### Plaque length measurement and antithrombotic therapy

The geometry of 144 plaques obtained from all 125 patients undergoing CE, including the length and diameter of plaques, was routinely measured by pathologists. We defined plaque length as the length of the coronary plaque obtained through CE. Plaque length-max and plaque diameter-max, which represent the plaque length and plaque diameter, respectively, in patients undergoing single-vessel CE and the maximum plaque length and maximum plaque diameter, respectively, in patients undergoing multivessel CE, were calculated. Systemic heparinization was initiated post-surgery to avoid thromboembolic complications or early occlusion of the coronary arteries. When the postoperative bleeding rate was <50 mL/h, heparin infusion was initiated 4 h post-surgery. Aspirin (100 mg) and clopidogrel (75 mg) were administered daily starting on postoperative day 1 (16).

### Assessment of the SYNTAX score and EUROScore II

The SYNTAX score was calculated for individual arteries and lesions using an online calculator (http://www.syntaxscore.com/calculator/start.htm). The EUROScore II was calculated for each patient using an online calculator (http://www.euroscore.org/calc.html). Both SYNTAX score and EUROScore II were conducted by two cardiologists (MG, HL) who were blinded to patient data.

### Acute PMI analysis and perioperative complications

Two cardiologists (MG, HL) determined PMI occurrence according to the plasma TNI levels, electrocardiograms, and echocardiography results (17). PMI was defined as an elevation of TNI values >10× the 99th percentile upper reference limit within 7 days of surgery, in patients together with either (a) new pathologic Q waves on the electrocardiogram or (b) new abnormal segment wall motion of the ventricular wall on echocardiography (17). Arrhythmia was defined as the occurrence of ventricular tachycardia, fibrillation, or atrial fibrillation within 7 days post-surgery.

### Coronary excision, pathology, and immunohistochemistry

With the patients' consent, we performed additional analysis of pathological features in 37 coronary plaques obtained from 27 patients. Immunohistochemical staining was performed for CD68, osteopontin, and osteocalcin, and the average optical values were assessed. Plaque pathological features were determined from the hematoxylin and eosin slides according to the American Heart Association classification (18). The selected specimens were decalcified in ethylenediaminetetraacetic acid (after being stored in 4% paraformaldehyde for 24 h), embedded in paraffin, and sawed into 5-mm-thick sections for the analysis of their morphological characteristics. Hematoxylin and eosin (H&E) staining was performed. Immunohistochemical staining for CD68 (mouse anti-human ab955, Abcam, Cambridge, UK) and osteopontin (rabbit anti-human ab214050) was performed, and the average optical values were assessed, as described below. Slides were scanned using Leica Biosystems software (Germany) and assessed using CaseViewer software (Pannoramic MIDI, 3D HISTECH, Hungary). Masson's trichrome histochemical staining (Sigma Aldrich, St. Louis, MO) was performed for collagen and fibrosis analysis.

The H&E slides were analyzed by two experienced pathologists. Morphological features, including a large necrotic core, thin fibrous cap, loose fibroelastic tissue, intra-plaque hemorrhage, calcification, and microcalcifications, were identified according to the American Heart Association classifications.

The average optical value analysis of CD68 and osteopontin was performed using Image-pro plus 6.0 software (Media Cybernetics, Inc., Rockville, MD). We randomly screenshotted three 400-fold fields of each slice, carefully ensuring consistent background lighting in all photographs. A brownish yellow color was selected as the unified standard for evaluating the positive expression of all photographs; the integral optical density and pixel area of each photograph were determined. The average optical value is expressed as integral optical density/pixel area; a large average optical value indicates a great positive expression level.

### Statistical analyses

Statistical analyses were performed using SPSS software (version 25, SPSS, Inc., Chicago, IL). Data were tested for normality using the Shapiro-Wilk test. Continuous variables with a normal distribution are presented as means ± standard deviations; non-normal variables are reported as medians [interquartile ranges (IQRs)]. Data were compared using a two-sample *t*-test or Mann–Whitney *U* tests. Categorical variables are presented as absolute numbers (percentages) and were compared using a *χ*^2^ test (with a Yates correction or Fisher exact test for smaller sample sizes).

Univariate and multivariable logistic regression analyses (forced entry method) were performed to assess the association between plaque length and PMI occurrence. Odds ratios (ORs) and 95% confidence intervals (CIs) were estimated using logistic regression analyses. In addition, univariate linear regression analyses (forced entry method) were performed to assess the association between preoperative plasma biomarker levels, plaque length, and all pathological features. Beta and 95% CIs were estimated using linear regression analyses. Receiving operator curves (ROC) were calculated for the prediction of PMI based on plaque length and the optimal plaque length cut-off value for predicting PMI was determined. A two-sided *p* value*<*.05 was considered statistically significant.

## Results

### Patient characteristics

**Figure 1:**
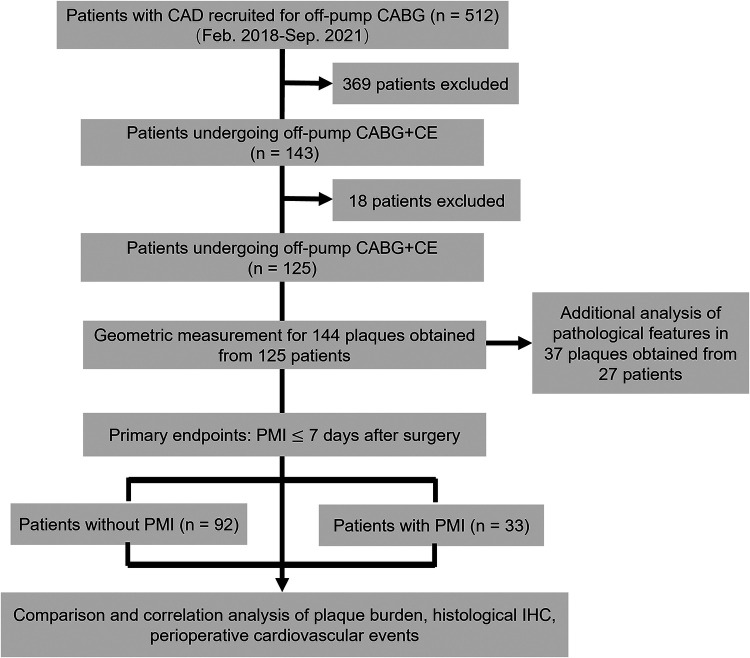
Study design flowchart. CAD, coronary artery disease; CABG, coronary artery bypass grafting; CE, coronary endarterectomy; IHC, immunohistochemistry; PMI, postoperative myocardial infarction.

This study recruited 143 patients with DCAD. Eleven patients with a recent MI, one with malignancy, and six with chronic hepatitis were excluded (Figure 1). Accordingly, the final study population consisted of 125 patients who had undergone CE (102 male; mean age: 61.9 ± 8.3 years), of whom 107 (85.6%) underwent single-vessel CE and 18 (14.4%) underwent multivessel CE (Table 1). Thirty-three patients (26.4%) experienced PMI, including three (9.1%) with PMI_anterior_, four (12.1%) with PMI_lateral_, 23 (69.7%) with PMI_inferior_, one (3.0%) with both PMI_anterior_ and PMI_lateral_, and two (6.1%) with both PMI_anterior_ and PMI_inferior_.

**Table 1:** Distribution of 125 patients and 144 vessels undergoing CE.

	LAD with CE	Diag with CE	OM with CE	PDA with CE	Patients (vessels) undergoing CE
Single-vessel (*n* = 107)	10	18	9	70	107 (107)
Multivessel	9	–	–	9	9 (18)
2 (*n* = 6)	–	–	3	3	3 (6)
3 (*n* = 4)	–	2	2	–	2 (4)
4 (*n* = 4)	–	2	-	2	2 (4)
5 (*n* = 2)	1	1	–	–	1 (2)
6 (*n* = 3)	1	–	1	1	1 (3)

CE, coronary endarterectomy; Diag, diagonal branch; LAD, left anterior descending artery; OM, obtuse marginal artery; PDA, posterior descending artery.

### Coronary plaque length and PMI

**Figure 2:**
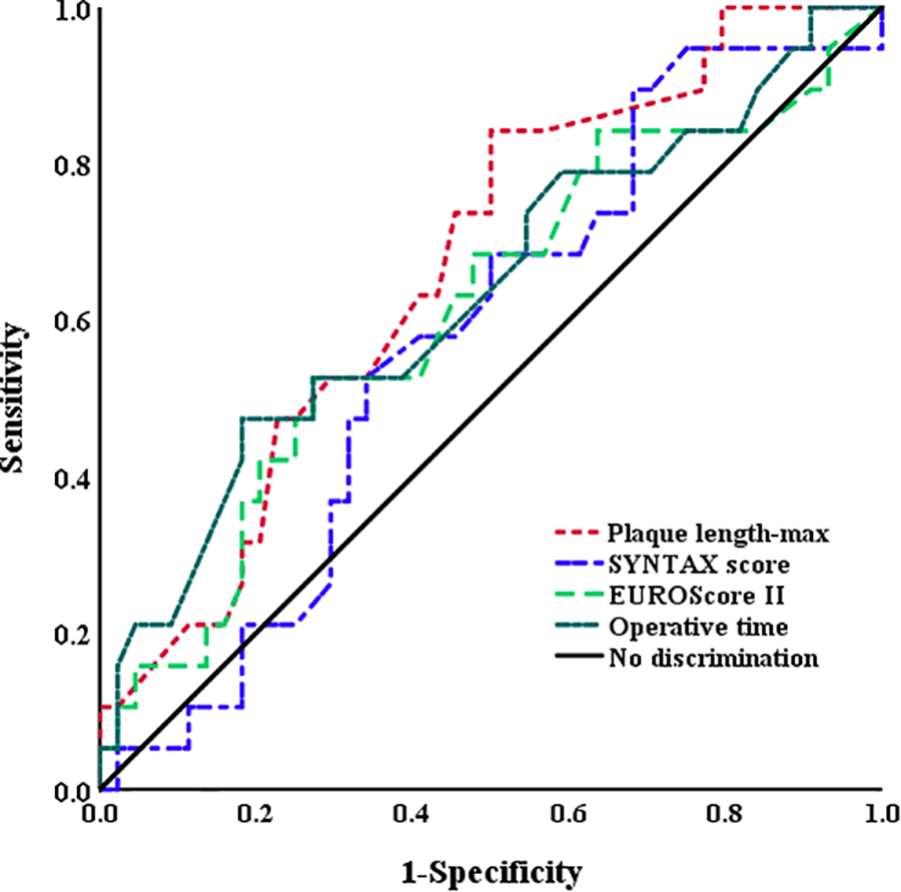
Receiving operator curves using plaque length-max, SYNTAX score, EUROScore II, and operative time for the identification of PMI. The AUC for the plaque length-max (0.67) was higher than SYNTAX score (0.57), EUROScore II (0.61), and operative time (0.64). AUC, area under curve; PMI, postoperative myocardial infarction.

Plaque length-max was significantly higher in patients who experienced PMI than in those who did not (2.4 ± 1.5 vs. 1.6 ± 0.9, *p* = .001) (Table 2). ROC curves analyses were constructed to evaluate the predictive values of plaque length-max, SYNTAX score, EUROScore II, and operative time for the identification of PMI (Figure 2). A threshold plaque length-max of 1.15 cm was a significant predictor of total PMI (area under the curve: 0.67; sensitivity 87.9%; specificity 59.8%; *p* = .005). The curve area of SYNTAX score, EUROScore II, and operative time were 0.57 (*p* = 0.353), 0.61 (*p* = 0.166), and 0.64 (*p* = 0.089), respectively. After adjusting for confounding factors of age, sex, body mass index (BMI), patients with plaque length-max ≥1.15 had a >5-fold increase in PMI [odds ratio (OR) = 5.89; *p* = .002]. Moreover, multivariate logistic regression analyses revealed that CE of the posterior descending artery had no significant correlation with PMI_inferior_ occurrence (OR = 3.05; *p* = .06). Patients with plaque length-max ≥ 1.15 had a > 5-fold increase in PMI_inferior_ after adjusting for confounding factors (OR = 5.84; *p* = .026) (Table 3).

**Table 2:** Distribution of all clinical parameters in patients with and without postoperative myocardial infarction.

Characteristics	All patients (*n* = 125)	Patients with PMI (*n* = 33)	Patients without PMI (*n* = 92)	*p* value
**Preoperative parameters**				
Mean age (years)	62.9 ± 8.3	61.8 ± 7.5	62.0 ± 8.6	.91
Male sex, *n* (%)	102 (81.6)	29 (87.9)	73 (79.3)	.43
Weight (kg)	72.7 ± 10.5	75.2 ± 11.4	71.9 ± 10.1	.13
BMI (kg/m^2^)	25.5 ± 3.1	25.7 ± 3.1	25.5 ± 3.1	.75
SYNTAX score	57.1 ± 23.2	59.6 ± 19.6	56.2 ± 24.7	.59
EUROS core II (%)	2.6 (2.0–3.8)	2.9 (2.3–4.2)	2.6 (1.9–3.7)	.21
hsCRP (mg/L)	1.5 (0.6–4.1)	1.7 (0.8–4.9)	1.3 (0.6–3.8)	.28
HDL-C (mmol/L)	1.0 ± 0.3	1.0 ± 0.4	1.0 ± 0.3	.48
LDL-C (mmol/L)	2.4 ± 1.0	2.3 ± 0.8	2.5 ± 1.0	.34
TG (mmol/L)	1.8 ± 1.0	1.7 ± 0.9	1.8 ± 1.1	.57
Glu (mmol/L)	6.3 ± 2.1	6.8 ± 2.5	6.2 ± 1.9	.12
CCR (mL/min)	91.4 ± 31.3	87.5 ± 31.0	92.9 ± 31.6	.40
IL6 (pg/mL)	7.2 ± 7.9	9.7 ± 13.8	6.3 ± 4.5	.21
TNF (pg/mL)	8.8 (7.4–10.8)	9.7 (8.4–12.0)	8.3 (7.3–10.8)	.23
Diabetes, *n* (%)	48 (38.4)	15 (45.5)	33 (35.9)	.41
Hypertension, *n* (%)	87 (69.6)	23 (69.7)	64 (69.6)	1.00
Stroke, *n* (%)	19 (15.2)	3 (9.1)	16 (17.4)	.40
Smoker, *n* (%)	77 (61.6)	22 (66.7)	55 (59.8)	.54
History of PCI, *n* (%)	21 (16.8)	4 (12.1)	17 (18.5)	.59
**Intraoperative off-pump CABG + CE parameters**				
CEs (*n*)	1.0 (1.0–1.0)	1.0 (1.0–1.0)	1.0 (1.0–1.0)	.84
Single-CE, *n* (%)	107 (85.6)	28 (84.8)	79 (85.9)	1.00
CE-LAD, *n* (%)	21 (16.8)	8 (24.2)	13 (14.1)	.19
CE-OM, *n* (%)	15 (12.0)	2 (6.1)	13 (14.1)	.35
CE-PDA, *n* (%)	85 (68.0)	23 (69.7)	62 (67.4)	1.00
Plaque length-max (cm)	1.8 ± 1.2	2.4 ± 1.5	1.6 ± 0.9	.001*
Plaque diameter-max (cm)	0.2 (0.2–0.2)	0.2 (0.2–0.2)	0.2 (0.1–0.2)	.62
Plaque length-max ≥1.15 (*n*)	80 (64.0)	29 (87.9)	51 (55.4)	.001*
Grafts (*n*)	4.0 (4.0–4.0)	4.0 (4.0–4.0)	4.0 (4.0–4.0)	.90
PI-LAD, *n* (%)	2.3 ± 0.8	2.2 ± 0.9	2.4 ± 0.8	.21
PI-Diag, *n* (%)	2.7 ± 0.9	2.6 ± 1.0	2.7 ± 0.8	.62
PI-OM, *n* (%)	2.3 ± 0.9	2.4 ± 0.8	2.3 ± 0.9	.66
PI-PDA, *n* (%)	2.1 ± 1.0	2.1 ± 1.0	2.1 ± 1.0	.82
Flow-LAD (mL/min)	26.0 (16.0–40.0)	27.0 (17.0–51.5)	25.0 (16.0–38.0)	.16
Flow-Diag (mL/min)	18.0 (12.0–32.3)	22.0 (14.0–33.5)	18.0 (11.0–32.0)	.45
Flow-OM (mL/min)	23.5 (13.3–41.0)	22.0 (9.0–45.0)	24.0 (14.5–39.5)	.63
Flow-PDA (mL/min)	24.0 (14.0–43.3)	25.0 (19.0–45.0)	23.0 (14.0–42.0)	.60
Operative time (min)	310.4 ± 57.2	327.3 ± 69.4	304.4 ± 51.3	.048*
**Postoperative complications**				
TNI (ng/mL)	1.3 (0.6–4.9)	2.2 (0.9–13.9)	1.1 (0. 6–2.7)	.009*
Arrhythmia (%)	25 (20.2)	7 (21.2)	18 (19.8)	1.00
All-cause mortality (%)	4 (3.2)	4 (12.1)	0 (0)	.004*
Cerebrovascular disease (%)	4 (3.2)	1 (3.0)	3 (3.3)	1.00
IABP (%)	25 (20.0)	11 (33.3)	14 (15.2)	.040*
ECMO (%)	4 (3.2)	3 (9.1)	1 (1.1)	.06
ICU stay (h)	23.0 (20.0–47.5)	41.0 (21.0–104.0)	22.0 (20.0–43.0)	.009*
LOS (days)	19.0 (14.0–24.0)	23.0 (15.0–27.5)	17.0 (14.0–22.8)	.030*
Hospitalization costs (10,000 RMB)	13.5 (12.8–16.1)	15.3 (13.3–23.6)	13.4 (12.6–14.8)	.002*

BMI, body mass index; CABG, coronary artery bypass grafting; CCR, creatinine clearance rate; CE, coronary endarterectomy; hsCRP, high-sensitivity C-reactive protein; Diag, diagonal branch; ECMO, extracorporeal membrane oxygenation; Glu, blood glucose; HDL-C, high-density lipoprotein cholesterol; IABP, intra-aortic balloon pump; ICU, intensive care unit; IL, interleukin; LAD, left anterior descending artery; LDL-C, low-density lipoprotein cholesterol; LOS, length of stay; OM, obtuse marginal artery; PCI, percutaneous coronary intervention; PDA, posterior descending artery; PI, perfusion index; PMI, postoperative myocardial infarction; TG, triglyceride; TNF, tumor necrosis factor; TNI, troponin I. Data are presented as medians (25th–75th percentile), *n* (%), or means ± standard deviations.

* *p < *.05 was considered significant.

**Table 3:** Association between PMI, PMI_anterior_, PMI_inferior_, and clinical parameters in univariate and multivariable logistic regression models.

	Covariate	Model 1		Model 2	
		OR (95% CI)	*p*	OR (95% CI)	*p*
**PMI**	CEs (*n*)	1.30 (0.48–3.53)	.60	1.28 (0.47–3.47)	.63
(*n* = 33)	Plaque length-max (cm)	1.74 (1.20–2.53)	.004*	1.77 (1.20–2.62)	.004*
	Plaque length-max ≥1.15	5.83 (1.90–17.92)	.002*	5.89 (1.90–18.27)	.002*
	SYNTAX score	1.01 (0.98–1.03)	.59	1.01 (0.98–1.03)	.58
	EUROScore II (%)	1.13 (0.93–1.36)	.22	1.20 (0.97–1.49)	.09
**PMI_anterior_**	CE-LAD (*n*)	0.99 (0.11–8.94)	.99	0.92 (0.10–8.64)	.95
(*n* = 6)	Plaque length-max (cm)	1.95 (0.76–4.96)	.16	–	–
	Plaque length-max ≥1.15	–	–	–	–
**PMI_inferior_**	CE-PDA (*n*)	2.95 (0.94–9.28)	.06	3.05 (0.95–9.86)	.06
(*n* = 25)	Plaque length-max (cm)	1.71 (1.11–2.63)	.015*	1.72 (1.09–2.70)	.019*
	Plaque length-max ≥1.15	6.09 (1.30–28.44)	.022*	5.84 (1.24–27.54)	.026*

BMI, body mass index; CE, coronary endarterectomy; CI, confidence interval; LAD, left anterior descending artery; OR, odds ratio; PDA, posterior descending artery; PMI, postoperative myocardial infarction. Model 1: univariate model. Model 2: adjusted for age, sex (male, 1; female, 0), and body mass index.

* *p* < .05 was considered significant.

PMI significantly increased TNI (2.2 [0.9–13.9] ng/mL vs. 1.1 [0.6–2.7] ng/mL; *p* = .009), all-cause mortality (4 [12.1] vs. 0 [0]; *p* = .004), use of an intra-aortic balloon pump (IABP) (11 [33.3] vs. 14 [15.2]; *p* = .040), rate of intensive care unit (ICU) stay (41.0 [21.0–104.0] h vs. 22.0 [20.0–43.0] h; *p* = .009), length of stay (LOS) (23.0 [15.0–27.5] h vs. 17.0 [14.0–22.8] h; *p* = .030), and hospitalization cost (15.3 [13.3–23.6] 10,000 RMB vs. 13.4 [12.6–14.8] 10,000 RMB; *p* = .002) (Table 2). There was no significant difference in serum biomarkers, SYNTAX score, EUROScore II, or intraoperative parameters between patients with and without PMI (all *p* > .05) (Table 2).

### Clinical variables, immunobiological examination, and plaque length

As shown in Table 4, linear regression analysis revealed that IL-6 was significantly correlated with plaque length-max (Beta = 0.32: *p* = .028), whereas the other clinical variables were not (all *p* > .05).

**Table 4:** Association between clinical parameters and plaque length-max in linear regression model.

Clinical parameters	Beta (95% CI)	*p* value
Mean age (years)	0.07 (−0.02–0.04)	.45
Male sex (%)	−0.03 (−0.64–0.46)	.74
Weight (kg)	0.02 (−0.02–0.02)	.83
BMI (kg/m^2^)	0.07 (−0.04–0.09)	.45
SYNTAX score	0.03 (−0.01–0.01)	.85
EUROScore II (%)	0.08 (−0.06–0.15)	.38
hsCRP (mg/L)	−0.04 (−0.02–0.01)	.65
HDL-C (mmol/L)	0.06 (−0.45–0.87)	.53
LDL-C (mmol/L)	−0.10 (−0.35–0.08)	.23
TG (mmol/L)	0.02 (−0.18–0.22)	.81
Glu (mmol/L)	−0.04 (−0.12–0.08)	.69
CCR (mL/min)	−0.09 (−0.01–0.00)	.33
IL6 (pg/mL)	0.32 (0.01–0.10)	.028*
TNF (pg/mL)	0.10 (−0.01–0.02)	.53
Diabetes	0.07 (−0.25–0.60)	.42
Hypertension	0.15 (−0.07–0.83)	.10
Stroke	−0.01 (−0.61–0.55)	.92
Smoker	0.03 (−0.36–0.50)	.74
History of PCI	−0.04 (−0.67–0.44)	.69

BMI, body mass index; CCR, creatinine clearance rate; hsCRP, high-sensitivity C-reactive protein; Glu, blood glucose; HDL-C, high-density lipoprotein cholesterol; IL, interleukin; LDL-C, low-density lipoprotein cholesterol; PCI, percutaneous coronary intervention; TG, triglyceride; TNF, tumor necrosis factor.

* *p < *.05 was considered significant.

**Figure 3:**
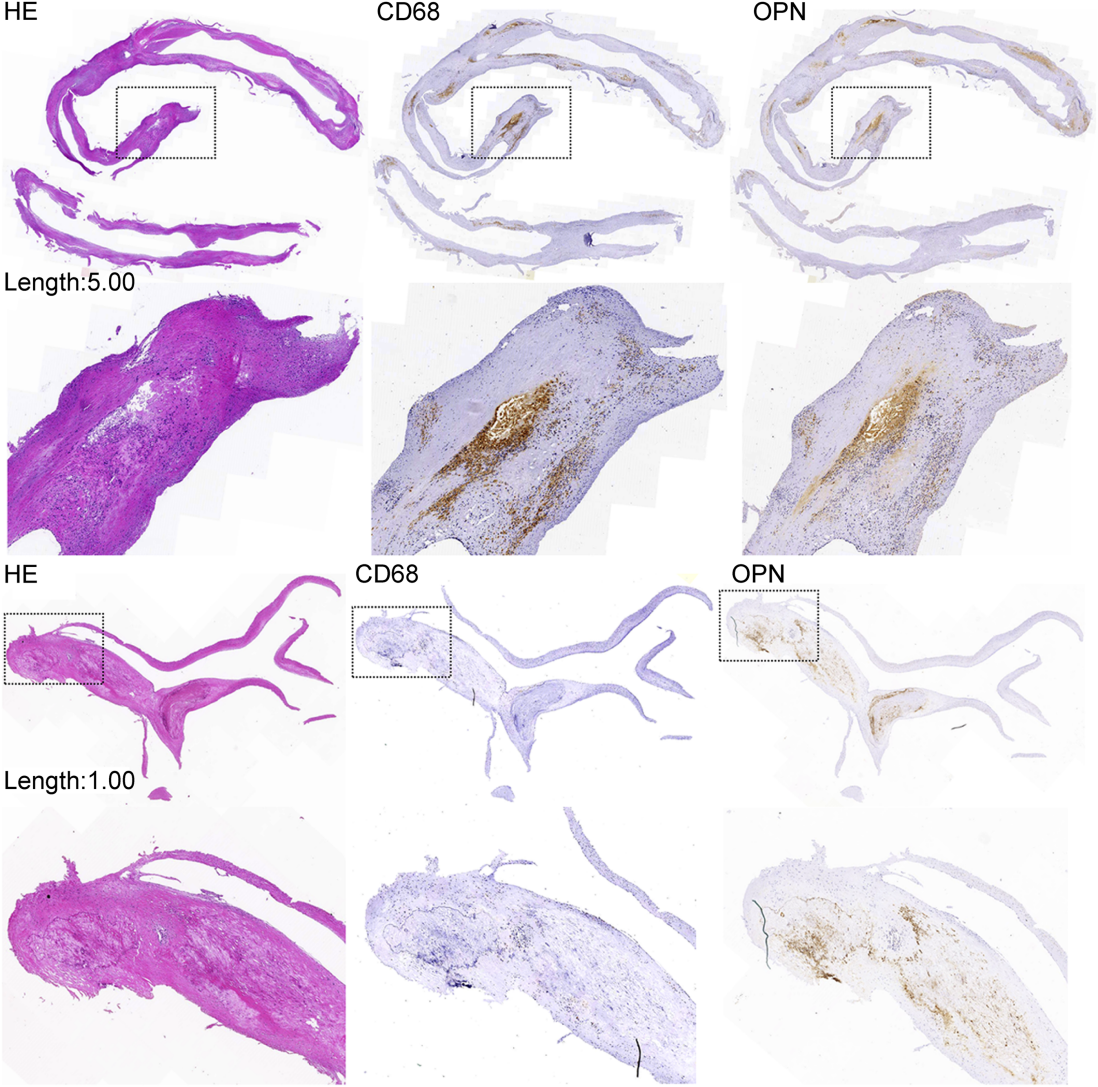
Coronary plaque length and immunochemical examination. The expression of OPN in plaques of length ≥1.15 cm is increased and correlated with increased CD68 expression. The expression of OPN in plaques of length <1.15 cm is decreased and correlated with decreased CD68 expression. The expression of OPN was also increased in microcalcifications adjacent to the initial calcification and at the margins of large macrocalcifications. HE, hematoxylin and eosin; OPN, osteopontin.

The average length of all 37 plaque specimens obtained through CE was 1.25 (IQR: 1.00–2.00) cm. Linear regression analysis revealed that CD68 (Beta = .34; *p* = .045), osteopontin (Beta = 0.43; *p* = .008), and calcium score (*r* = .52; *p* = .001) were significantly correlated with plaque length-max (Table 5). Patients with plaque length-max ≥1.15 cm had increased average optical values of osteopontin co-localized in areas with higher CD68 expression. In plaques of length <1.15 cm, a portion of osteopontin was localized in microcalcifications adjacent to the initial calcification and margins of large macrocalcifications (Figure 3).

**Table 5:** Association between histopathology features and plaque length in 37 coronary specimens in linear regression model.

Histopathology features	Beta (95% CI)	*p* value
CD68 (AO)	0.34 (0.00–0.00)	.045*
OPN (AO)	0.43 (4.24–26.60)	.008*
Calcium score	0.52 (0.02–0.05)	.001*
Large necrotic core	0.31 (−0.05–1.86)	.06
Thin-fibro cap	0.24 (−0.28–1.63)	.16
Loose fibroelastic tissue	0.22 (−0.31–1.59)	.18
Intra-plaque hemorrhage	−0.17 (−1.73–0.60)	.33
Microcalcification	0.13 (−0.89–1.93)	.46
Calcification	0.17 (−0.54–1.69)	.30

AO, average optical value; OPN, osteopontin; CI, confidence interval.

* *p < *.05 was considered significant.

## Discussion

**Figure 4:**
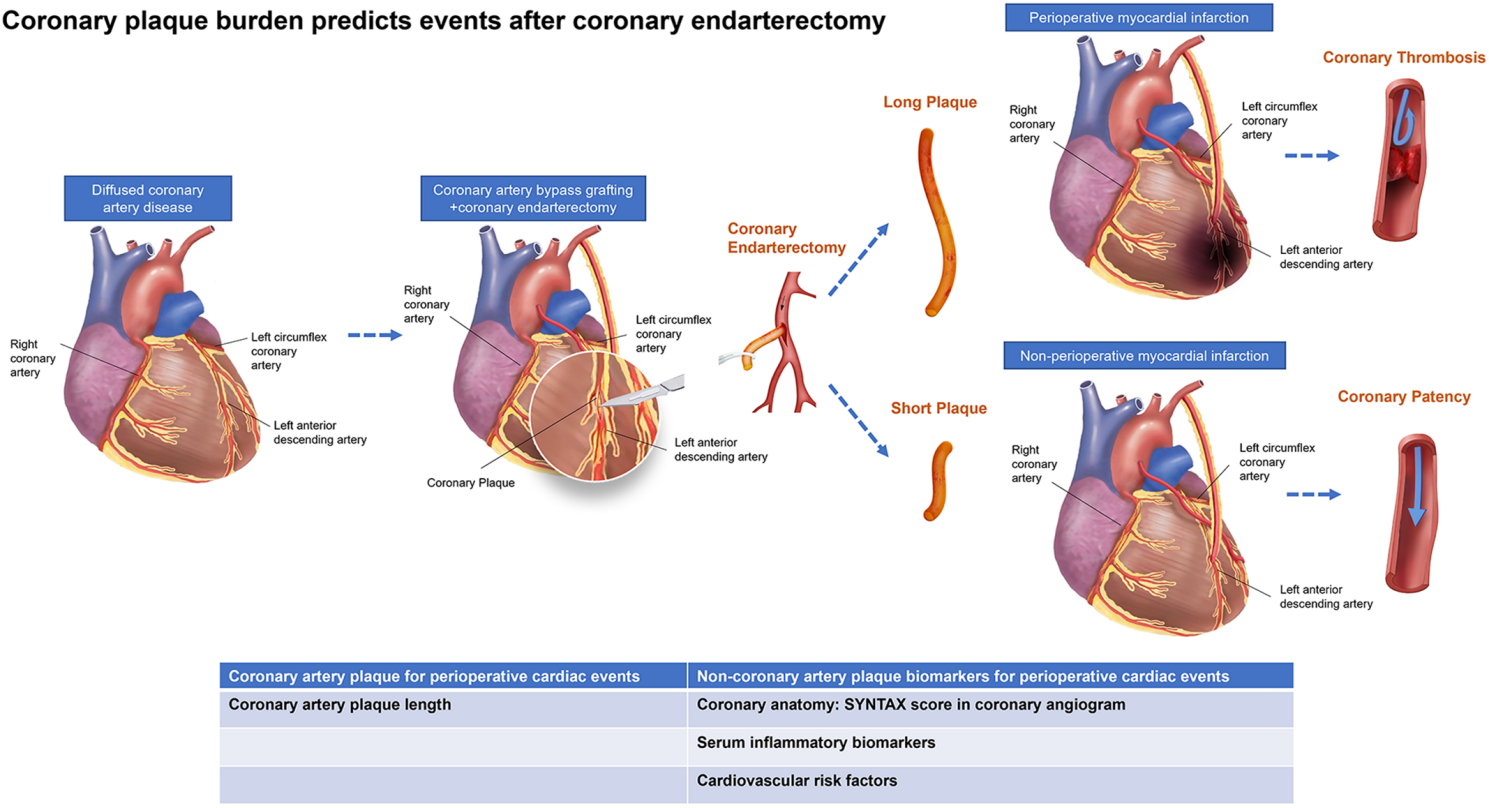
Central illustration. Schematic illustration of the occurrence of adverse events shows vascular occlusions after off-pump coronary artery bypass grafting compatible with coronary endarterectomy in patients with diffused coronary artery disease. Multifactorial validation on both coronary arteries’ anatomy, plaque morphology, and serum biomarkers may yield further insights into the interplay of alternative morphological and molecular processes, including coronary stenosis, inflammation, osteogenesis, and metabolic processes.

We analyzed the predictive value of plaque length for occurrence of PMI after off-pump CABG with adjunctive CE. We found that plaque length-max correlated with PMI occurrence and a plaque length-max threshold of 1.15 cm was an independent predictor for PMI. Furthermore, we also found a correlation between focal plaque length and IL-6, a systemic inflammatory biomarker, and validated the correlation between plaque length *in vivo* and plaque histopathologic features *in vitro*. To the best of our knowledge, this is the first study to predict perioperative cardiovascular events by coronary plaque burden in patients who underwent off-pump CABG with adjunctive CE (Figure 4).

### Effect of CE for DCAD

CABG is the standard treatment strategy for complete revascularization in patients with complex CAD and incomplete revascularization is a critical negative factor influencing long-term mortality and morbidity (2, 19). DCAD accounts for 25% of patients with complex CAD, and complete revascularization is rarely achieved by CABG alone in these patients (20, 21). Therefore, CE is recommended as an adjunct to CABG for DCAD patients to achieve complete revascularization via the removal of coronary plaques in the target vessels if complete revascularization could not be achieved (5, 15, 22). However, a consequent rise in PMI occurrence and 30-day outcomes is worse in patients undergoing CABG adjunct with CE than in those undergoing CABG alone (23, 24). PMI rate was 24.7% in our study, which was higher than the rate of 2.8% in patients undergoing CABG alone reported in previous literature (6).

Compared with closed-CE, open-CE typically achieves complete plaque removal, prevents postoperative thrombotic occlusions, and reduces perioperative cardiac events, particularly in patients with DCAD in the left coronary descending artery (LAD) (7, 8, 25–27). Nevertheless, based on empirical data for patients with DCAD in other target arteries that are harder to access than the LAD, closed-CE is a good choice to achieve complete revascularization. In this study, all 125 patients underwent closed-CE, and a second CE was performed to remove the total distal end of the plaque if the graft patency of the distal target vessel or its side branches was insufficiently revascularized, as judged by the Veri Q flow meter. The prevalence of target vessels for CABG adjunct with CE was 16.8% in LAD, 18.4% in diagonal branches of the LAD, 12.0% in left circumflex, and 68.0% in posterior descending artery. This may explain why the incidence of total PMI, particularly PMI_inferior_, was higher in this study than in a previous study (28). Moreover, patients with PMI had higher rates of perioperative all-cause mortality and IABP procedure, longer ICU stay and LOS, and greater hospitalization costs than those without PMI. It indicated that the identification of high-risk patients who are more likely to develop PMI is very important.

### Prognostic value of plaque length

Intact coronary endothelium can produce vasoactive biomarkers to neutralize leukocyte adhesion and to inhibit platelet aggregation, consequently reducing inflammation and thrombosis in a target artery (29, 30). The poor short-term postoperative outcomes in our current study patients with DCAD undergoing CABG + CE may be due to endothelial cell injury, loss of cytoprotective factors, unsteady blood flow or shear stress, and distal residual lesions caused by closed-CE (12–14). Acute PMI, a key complication associated with CE failure, is also associated with thrombosis, which can be induced by endothelial lining removal (13, 14, 31). Theoretically, endothelium damage and distal residual lesions are correlated with the plaque length removed by CE. Surgeons always try to reduce endothelium damage by shortening plaque length during CE. However, there are no criteria for surgeons to determine the location and length of the CE. The key issue is that the association of plaque length with acute perioperative clinical outcomes remained unclear. We found that increased plaque length was significantly associated with PMI occurrence. Patients with plaque length-max ≥1.15 had a >5-fold increase in PMI and a >5-fold increase in PMI_inferior_. Therefore, surgeons should adequately assess the benefits and adverse effects of CE and preoperatively determine the location of the CE to achieve satisfactory clinical outcomes. Although the AUROC of plaque length-max in predicting PMI was 0.67, it was still more powerful in predicting events than other risk factors in this relatively rare sample.

### Role of serum systemic inflammatory biomarkers in plaque

The elevation of serum systemic inflammatory biomarkers post-surgery, including IL-6, tumor necrosis factor, and C-reactive protein, is correlated with PMI incidence. While the prognostic significance of serum biomarkers remains controversial (32–35). We found that preoperative serum biomarkers were not correlated with PMI occurrence. This suggests that serum biomarkers have limited early prognostic value due to a lack of organ specificity and less sensitivity for predicting postoperative cardiac events. Recent studies have found that plasma levels of sLOX-1 can predict fatal events at 1 year in ACS patients and JCAD can promote the formation of arterial thrombosis in STEMI patients by selectively regulating coagulation and fibrinolysis. Whereas, the power of sLOX-1 predicting cardiovascular events after CABG or JCAD preventing the formation of platelet thrombosis after CABG remains to be investigated (36, 37). IL-6 plays a central role as a mediator of the inflammatory response and is essential for the initiation and progression of the atherosclerotic process (38, 39). We found a linear correlation between IL-6 and plaque length-max. It suggested that IL-6 could reflect the severity of inflammation in total atherosclerotic plaques and can be considered as an indirect parameter for the prediction of PMI. In a previous intracoronary imaging study with optical coherence tomography (OCT) that investigated outcomes of patients with non-culprit OCT-defined vulnerable plaques, serum levels of C-reactive protein were not associated with intraplaque accumulation of inflammatory cells (OCT-defined macrophages). This is probably due to the more specific role of IL-6 compared with that of C-reactive protein. Furthermore, a positive association between intraplaque inflammatory cells and adverse clinical events was found in the paper by Gatto et al., further corroborating results obtained in the present study (40).

### Intimal osteopontin activity and inflammation in atherosclerotic plaque

Osteopontin is expressed in human atherosclerotic plaques and is strongly correlated with vulnerable plaques. Carotid plaque osteopontin and serum osteopontin levels have been reported to predict cardiovascular events (41, 42). Systemic inflammation biomarker (IL-6) may trigger IL-6 trans-signaling, contributing to the upregulation of osteopontin in macrophages (43, 44). Moreover, osteopontin expression is associated with macrophages and foam cells within atherosclerotic lesions (45). In our study, osteopontin expression in coronary plaques >1.15 cm in length was significantly increased. Osteopontin and CD68 also were correlated with plaque length. Thus, it could be explained that both systemic inflammation (IL-6) and regional inflammation (CD68) possibly contribute to plaque vulnerability and lead to coronary plaque progression. We postulated that, in patients with increased plaque length who underwent off-pump CABG + CE, the inflammatory microenvironment within the damaged coronary artery activates processes, such as thrombosis processes, subsequently leading to PMI, and it may affect the long-term cardiac events.

### Limitations

First, the sample size was relatively small; and it was not sufficient to evaluate the association between the CE location and local PMI. Second, recruiting a homogeneous group of patients with severe CAD may have biased the CABG indications. Third, contrast-enhanced coronary CT or coronary angiography should have been performed in patients with PMI to confirm the graft patency of affected vessels despite the fact that most patients with PMI had stable hemodynamics. Finally, a long-term follow-up study is warranted to validate the prognostic value of plaque length for long-term outcomes in these patients.

## Conclusions

Plaque length-max was an independent predictor for PMI occurrence in patients undergoing off-pump CABG + CE, which might be associated with systemic and regional plaque inflammation.

## Data Availability

The original contributions presented in the study are included in the article, further inquiries can be directed to the corresponding authors.
